# Characterisation and mapping of scattered radiation fields in interventional radiology theatres

**DOI:** 10.1038/s41598-020-75257-5

**Published:** 2020-10-30

**Authors:** M. Nowak, P. Carbonez, M. Krauss, F. R. Verdun, J. Damet

**Affiliations:** 1 CERN, European Organization for Nuclear Research, Geneva, Switzerland; 2 Institut of Radiation Physics, Lausanne University Hospital and University of Lausanne, Lausanne, Switzerland; 3 Department of Radiology, University of Otago, Christchurch, New Zealand; 4 Department of Interventional Radiology, Christchurch Hospital, Christchurch, New Zealand

**Keywords:** Health occupations, Applied physics, Techniques and instrumentation

## Abstract

We used the Timepix3 hybrid pixel detector technology in order to determine the exposure of medical personnel to ionizing radiation in an interventional radiology room. We measured the energy spectra of the scattered radiation generated by the patient during X-ray image-guided interventional procedures. We performed measurements at different positions and heights within the theatre. We first observed a difference in fluence for each staff member. As expected, we found that the person closest to the X-ray tube is the most exposed while the least exposed staff member is positioned at the patient’s feet. Additionally, we observed a shift in energy from head to toe for practitioners, clearly indicating a non-homogenous energy exposure. The photon counting Timepix3 detector provides a new tool for radiation field characterisation that is easier-to-use and more compact than conventional X-ray spectrometers. The spectral information is particularly valuable for optimising the use of radiation protection gear and improving dosimetry surveillance programs. We also found the device very useful for training purposes to provide awareness and understanding about radiation protection principles among interventional radiology staff.

## Introduction

Proper evaluation of worker exposure is a major issue and current challenge in radiation protection. The effectiveness of worker protection greatly depends on an understanding of the radiation field to which the staff is exposed. A radiation field can be described by fundamental physical quantities: the particle fluence and the energy spectrum. Besides physics, evaluating worker exposure is based on current knowledge of the biological effects of ionizing radiation. The concept of equivalent dose for any given organ and effective dose was introduced for risk management purposes as well as to establish annual dose limits^1^. The effective dose cannot be measured. The calculation of the effective dose integrates factors related to biological effects observed in radiobiological and epidemiological studies^2^.

The continuous improvement in understanding the underlying mechanisms of the health effects due to exposure to ionizing radiation motivates a regular re-evaluation of the annual limits.

In 2011, the International Commission on Radiological Radioprotection (ICRP) further restricted the annual eye lens dose limit, resulting in changes to the legal limits in Europe and Switzerland^2^. Before 2011, the annual dose limit for the eye lens was 150 mSv, this limit was set in order to prevent possible cataracts at the end of a career. It was agreed by consensus that if the annual effective dose limit was respected for the whole body it would also imply compliance to the eye lens dose limit^3^. The eye lens dose limit being relatively high, the probability of exceeding it was then quite low. Following a re-evaluation of the effects on the Hiroshima and Nagasaki cohort^4^, the limit was then lowered from 150 to 20 mSv^5^. The division of the limit by a factor of 7 significantly changes the stakes, as a large number of staff members may be over this limit. Because of the introduction of this more restrictive limit, a new dedicated operational quantity (H_p_(3)) was then proposed for managing eye lens exposure^6^. This prompted strong debate on the methods and means of measurements as well as calibration procedures for the measuring instruments.

The 2017 ICRP report also points out the need to revise the operational quantities for external exposure^7^. The ICRU sphere is not used anymore; direct conversion coefficients are instead given to the operational quantities from the fluence using anthropomorphic reference computational phantoms. In line with this new approach, we decided to characterise the radiation field in an interventional radiology (IR) theatre.

Our second reason for wanting to characterise the radiation field is related to the evolution of imaging systems. Medical imaging experts are permanently optimising the dose received by patients^8^, as well as using increasingly sophisticated imaging systems and improved image reconstruction algorithms. Additional filters are used to optimise the procedures (patient morphology, paediatric, etc.). These changes influence the energy spectrum used to image the patient’s body and thus the energy spectrum of the scattered radiation to which the medical staff is exposed. These changes are often accompanied by a decrease of the dose received by the hospital staff per procedure. Recent studies show that the development of more complex and longer procedures combined with the higher number of patients inevitably leads to an increase in exposure of hospital staff members^9^. Most procedures require staff to remain close to the patient during the image-guided procedures. A study by Sanchez et al*.*^10^ carried out on radiologists shows that more than 50% of them receive a dose higher than the authorized annual limit of 20 mSv, sometimes going up to monthly doses higher than 20 mSv on apron. This means it is important to establish an accurate dosimetry surveillance program in order to avoid underestimating worker dose. Knowledge of the spectrum to which the medical staff is exposed would enable a more accurate calculation of the dose, but would also make it possible to retrospectively know the dose received by an organ and this despite the evolution over time of the operational quantities for external exposure.

The spectral composition of X-ray radiation has been studied for a long time. Although many studies have been carried out based on computer simulations of the spectrum, very few are based on direct measurements. The first studies of the spectral composition of beams from an X-Ray tube date back to the mid-30’s^11,12^. The X-ray spectral information was first investigated by measuring attenuation curves. Forty years later, the first computer simulations on primary radiation were performed^13–15^. The first direct measurements of the spectrum were in 1969 by Peaple and Burt^16^, later followed by Seelentag and Panzer^17^ and Birch and Marshall^18^ using crystal scintillator detectors (NaI) or a semiconductor detector (Ge). The measurement set-up leads to cumbersome experimental conditions and involved strong constraints such as concomitant cooling or reducing the counting rate with a distance of several meters to the source and high collimation. In 1983, Castro et al*.*^19^ attempted to solve those problems, using a CdTe detector with a low detection efficiency. However, this kind of measurement requires long-term work along with a large number of corrections and is not suitable for rapid cumulative dose measurements for a procedure or a configuration. More recently, Miyajima et al.^20^ used a CdZnTe detector for spectra measurement, with results compatible with those obtained by Monte-Carlo (MC) simulations. The detector has several problems such as poor charge transport properties and requires complex calculated corrections for the spectral distortion.

Adapting a detector to a routine dosimetry application would imply that the device could be used regardless of the radiation field, the collimation, or the position in the radiation field. A very challenging point is the acquisition speed of the data. Missing data can lead to a wrong estimation of the photon fluence and dose. Another challenge to negotiate is making the detector small enough to be comfortable for the user to wear.

With the introduction of new technologies, the measurement of the energy spectrum in hospital theatres can provide optimised radiation protection safety for workers. Since 1997, the CERN Medipix team has been developing novel photon detectors that are able to provide spatial, temporal, and energy information for each incoming photon. This means that these detectors are able to measure the energy spectrum in real time. In this work, we used the Timepix3 detector to measure the energy spectrum of the scattered radiation field in an IR theatre. Compared to other spectral detector^16–20^, this detector has the advantage of coping with higher photon fluences and not requiring beam collimation with heavy pinhole shielding. It can be used at any distance from the radiation source in the scattered field and data are available instantaneously. This enabled us to compare the different spectra for different positions in the room, and for different heights. We then created a map of the scattered radiation field extracted directly from the energy spectra as received by a staff member around the X-ray tube.

## Materials and methods

### Timepix3 detector and software

The Timepix3 chip is a hybrid pixel detector (HPD) developed in the framework of the Medipix3 collaboration at CERN^21^, and is a latest version of the Timepix family^22^. We used the Timepix3 chip with a 500 μm thick silicon sensor produced and bump bonded by ADVACAM (Prague, Czech Republic)^23^. We choose a Si sensor layer despite a low absorption efficiency (4% detection at 60 keV versus 87% for a 500 µm CdTe sensor)^24^ as this is counterbalanced by a more stable Si response since the production of fluorescence photons is much higher in CdTe, from 80 to 90% versus less than 5% for Si. With a mean free path of fluorescence photons of 10 μm with Si and 110 μm with CdTe. The chip has a pixel matrix of 256 × 256 square pixels with 55 μm side and can process up to 40 Mhits/cm^2^/s. Each pixel sensor is linked to his own readout. A 500 nm aluminium layer covers the sensor. The sensor was biased at 300 V. The data driven mode was selected to collect information on arrival time, charge deposit, and the coordinates of each pixel hit by a photon. The charge deposit was calculated based on the time over threshold value. Each pixel was calibrated independently using radioactive sources and fluorescence peaks of different material foils^25^. We choose to analyze data with signal collected with single pixels only. The choice was made to eliminate additional uncertainties on energy calculation for photons generating a signal on several pixels (pixel sharing correction). Single pixels data do indeed represent only a fraction of all detected photons. The data have thus been normalized taking into account the ratio between the number of photons with single pixel and multiple pixel signals. The overall correction includes the detector efficiency as well as for angular variation, temperature, and light variation, since calibration condition can be different than measurement conditions^26^. Uncertainties have been calculated for energy calibration, pixel sharing, detector efficiency, position, angular variation, temperature, and light variation.

In this study, the Timepix3 chip was connected to the AdvadDAQ (Advacam, Prague, Czech Republic) readout system and data was collected using the Pixet software, version 1.5.0.714 (Advacam, Prague, Czech Republic).

### Measurement in interventional radiology theatre

**Figure 1:**
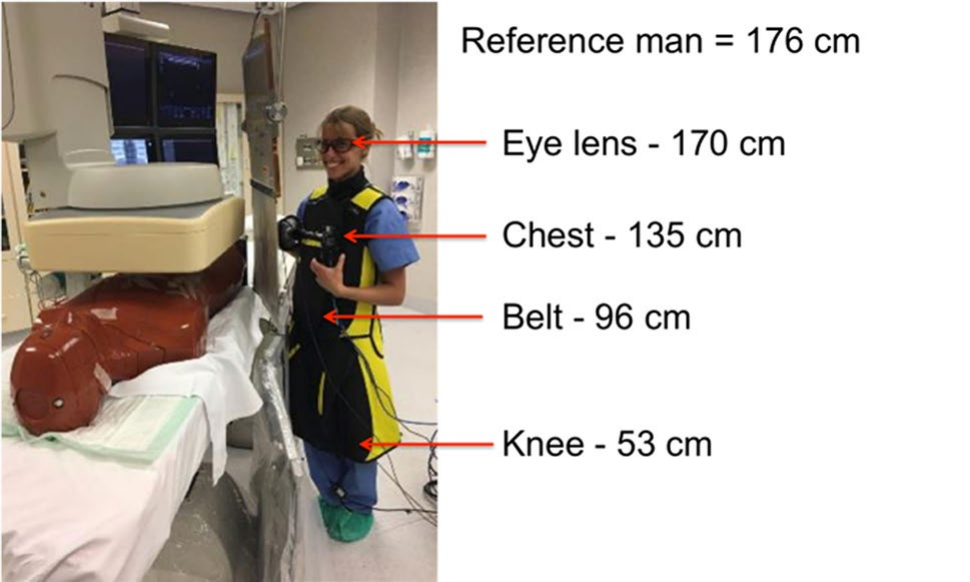
Measurements taken at four different heights in the IR theatre.

All measurements were carried out in an IR theatre at Christchurch Hospital—New Zealand, using an X-ray imaging system ALLURA XPER FD 20 (Philips, Hamburg, Germany). An anthropomorphic male phantom (RSD, Long Beach, CA, USA) was used to simulate a patient lying on the radiotransparent carbon fiber table. Energy spectra of the scattered radiation field were acquired around the table with the Timepix3 detector. Measurements were taken in a 3 m radius half-sphere centered on the abdomen of the phantom and at four different heights corresponding to the eye lens, chest, belt and knee, respectively 170 cm, 135 cm, 96 cm and 53 cm of a 1.76 m medical staff member (Fig. 1). The chosen protocol was the default set-up for pelvis/iliac examinations with an image acquisition rate of 3 frames per seconds (fps), a tube voltage of 74 kVp with a current of 12.0 mA, using the mode of standard dose for a reference patient (70 kg), leading to a filtration of 0.4 mm Cu + 1 mm Al. The cumulative time of measurements depended on the position in the room and ranged from 1 min for the closest positions to 4 min for the furthest away. Each data acquisition point was repeated 3 times; in this article we will present only the mean value of the three measurements for each position. All data have been normalized on 4 min measurement.

**Figure 2:**
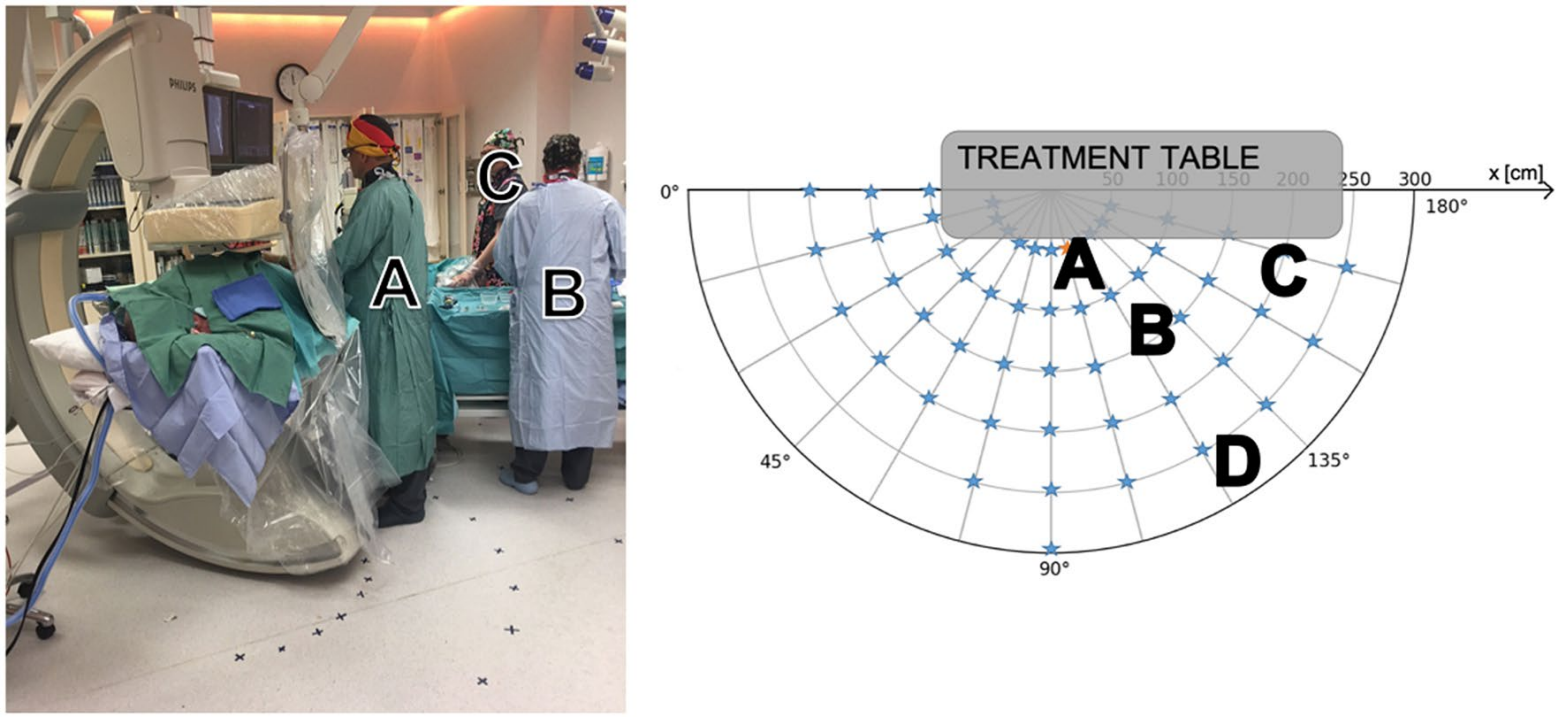
Left: positions of the different staff members in the IR room during procedure. Right: diagram of the different measurement positions (represented by red or blue stars) to create the scattered radiation map of the IR theatre.

A first series of measurements consisted in measuring the radiation field at the standard positions where the staff members stand during a routine procedure. After a period of observation and discussion with the team in place, we identified the four most common positions (see Fig. 2). Collective protection gear (suspended lead screen and lead table drape) were systematically used. We held the Timepix3 chip by hand above the lead apron, and we performed the measurements at the four heights successively in the four positions where the staff stand during the procedures, identified as A, B, C and D in the theatre on Fig. 2.

A second series of measurements consisted in measuring the energy spectra around the patient couch. Acquisitions were taken every 15°, on 180°, around the anthropomorphic phantom, every 50 cm (Fig. 2, right), for the four heights mentioned previously. The cumulative time of measurements depended on the position in the room and ranged from 1 min for the closest positions to 4 min for the furthest away. All data have been normalized on 4 min measurement. The Timepix3 chip was set on a metallic stand infusion holder. No specific means of protection were used. Moreover, the effect of the radiation protection gear (lead screen and lead table drape) was tested at a few positions in the room. On Fig. 2 right, each intersection between an arc and a line represents a position of measurement. The configuration of the room (walls, furniture) did not allow us to do all positions. Measurement points are represented by stars (blue or red). All measurements were performed after the surgery planned for the day. No measurement was done with a patient present in the theatre. These measurements were used to create a map of the theatre. Data has been corrected for pixel sharing and detection efficiency. Each spectrum obtained was segmented into energy bins, each multiplied with an energy-specific weighting factor. The values represented on the map are interpolated with a linear function between the measurement points.

### Ethical approval

We confirm that all the methods in this study have been applied in accordance with national guidelines and regulations. The consent of each participant was obtained prior to measurements. Each participant was informed and aware of the risks associated with ionizing radiation. Experimental protocols were approved by CERN Radiation Protection group.

A written consent to publish was obtained by all human participants involved in the study.

## Results

**Figure 3:**
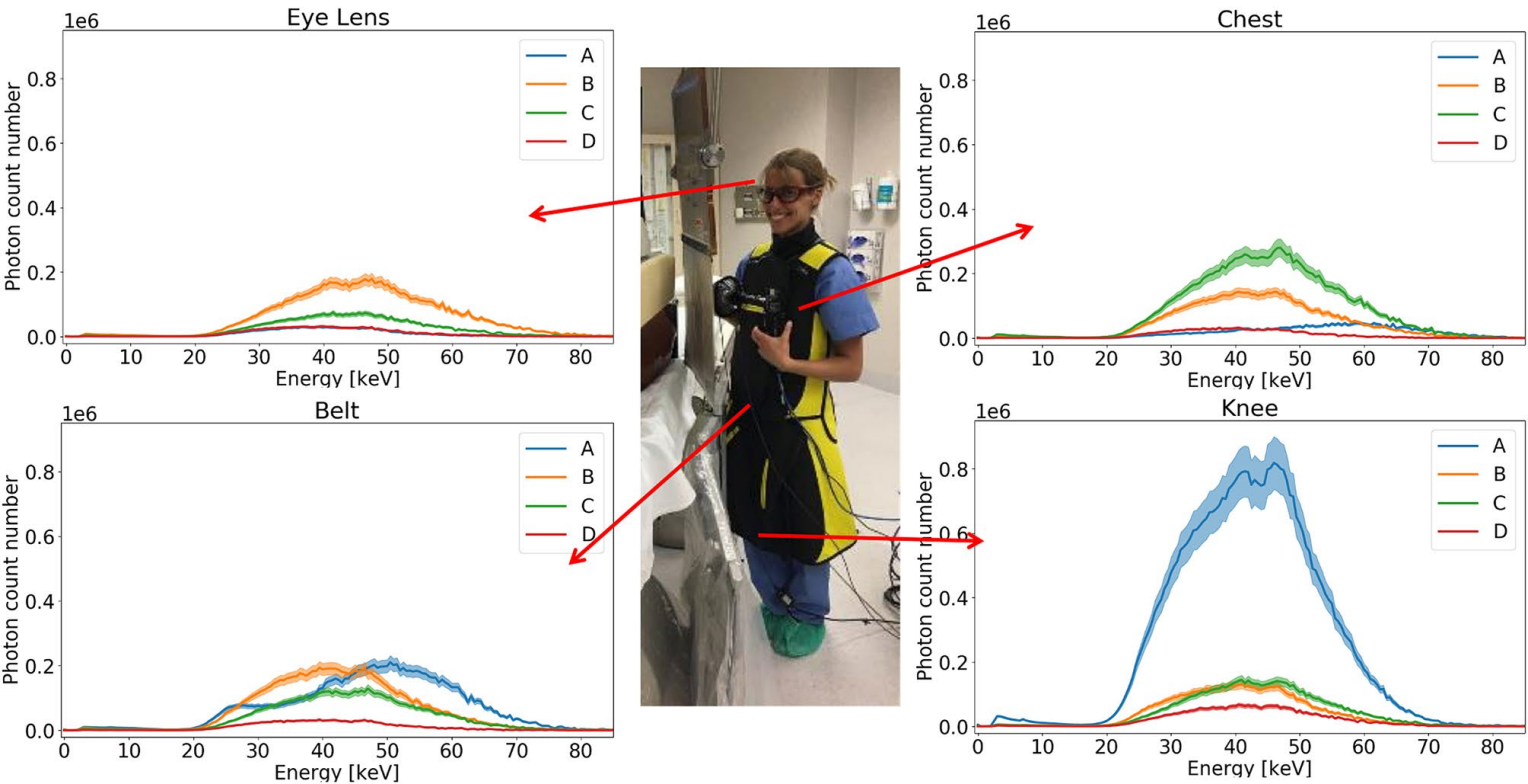
Energy spectra for each medical staff at a given height, with collective protections. Light curves represent the uncertainty.

**Figure 4:**
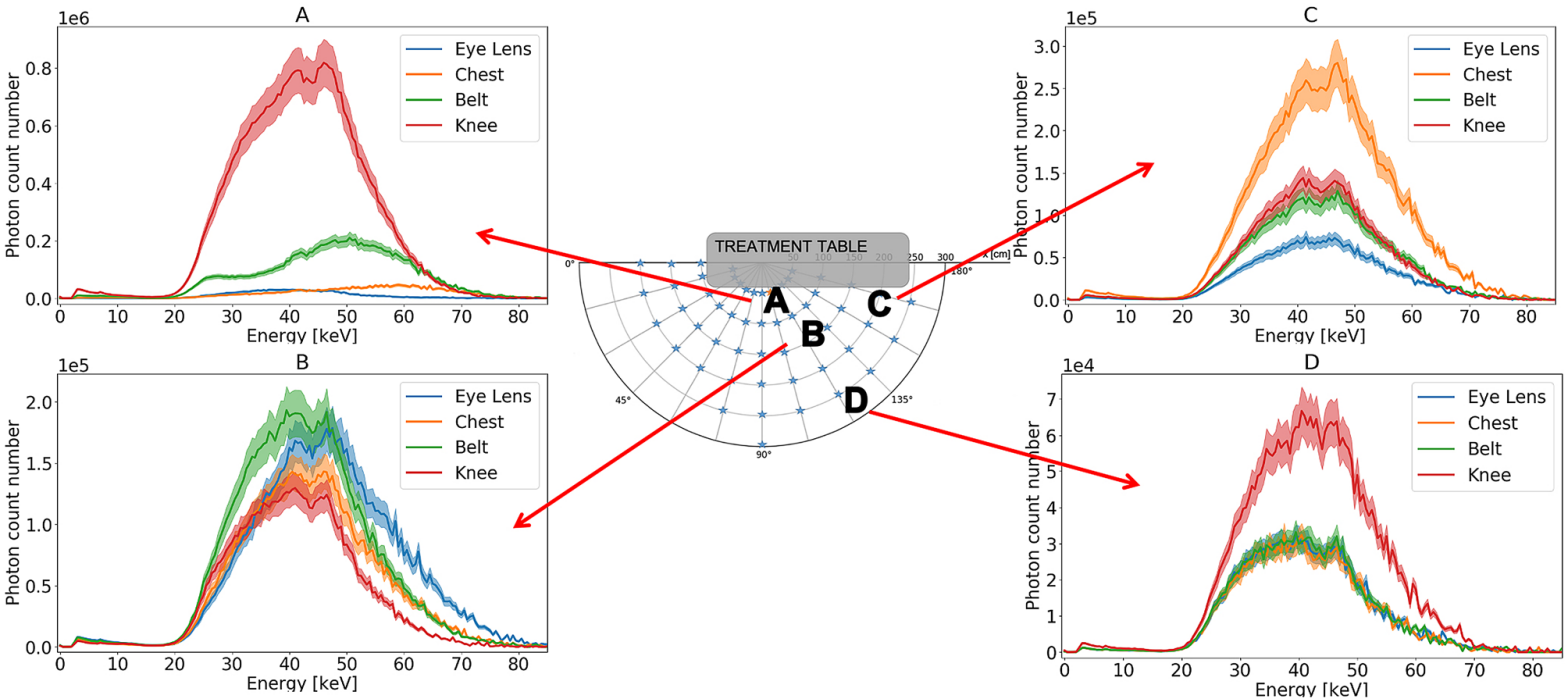
Energy spectra in absolute values for each height for a given person, with collective protections. Light curves represent the uncertainty.

Figures 3 and 4 show the energy spectrum measured on a person holding the Timepix3 chip at four heights in the four standard positions of the medical staff. Both figures present the same dataset, in a different way. Figure 3 presents the spectrum measured for a given height for each staff member. On Fig. 3 scales have been adapted to make the comparison easier. Figure 4 shows the energy spectrum measured at four heights for a given staff member. Scales are position dependent.

**Figure 5:**
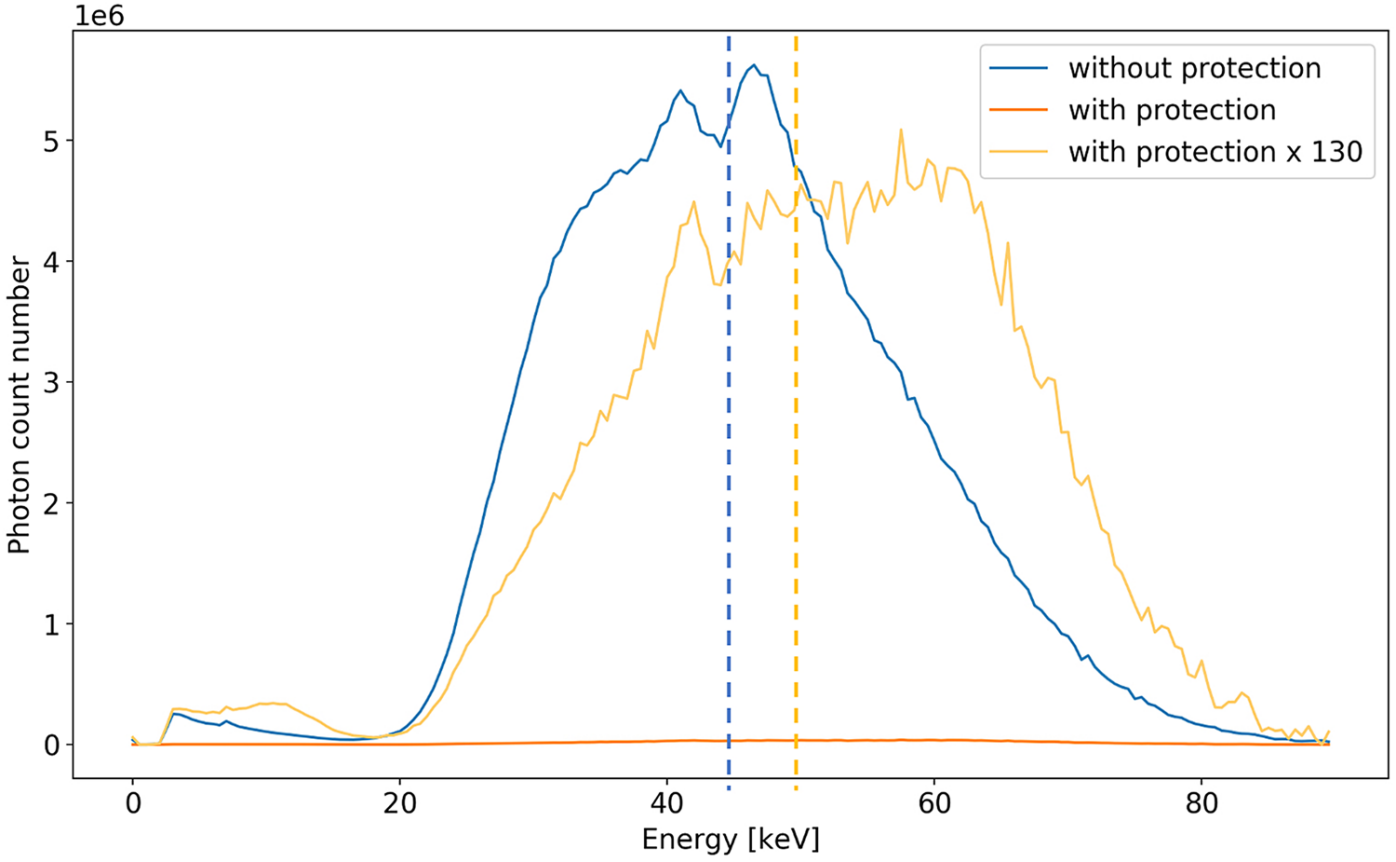
Energy spectra at 50 cm of the X-ray tube for an angle of 105° at 135 cm of height. Blue line represents the spectra without radiation protection gear, the orange line represents the spectra obtained with a lead screen and a lead table shield, the yellow line represents the data of the orange spectrum multiplied by 130. The dotted line represents the average energy.

**Figure 6:**
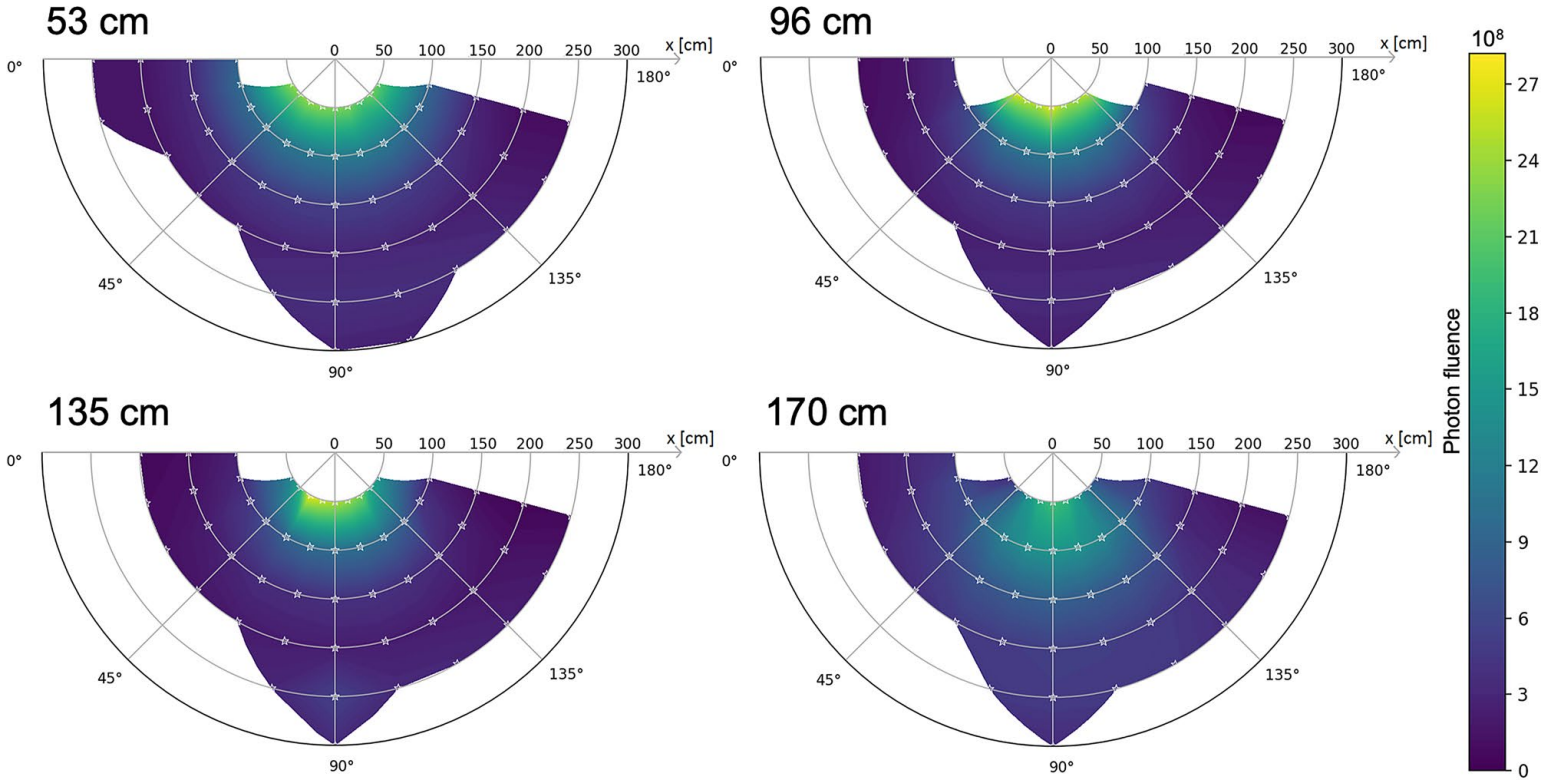
Colour maps of the photonic fluence in the theatre in the horizontal place at four heights, without collective protections.

**Figure 7:**
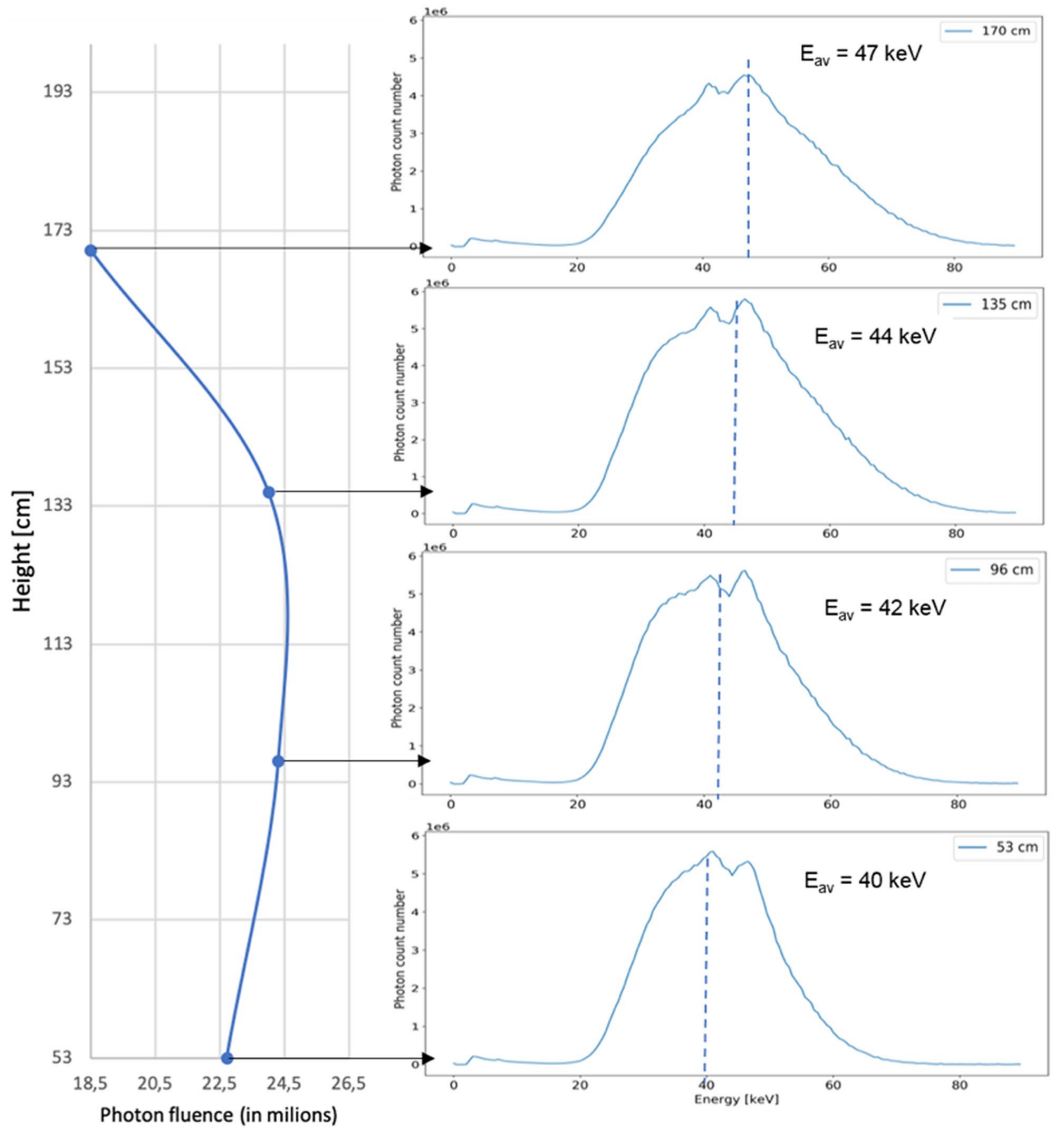
On the left part, the fluence multiplied by a factor energy-dependent as a function of the height, on the right part the energy spectra corresponding for each different height (from top to bottom: 170 cm, 135 cm, 96 cm, 53 cm). E_av_ represent the average energy.

Figure 5 shows the different energy spectra obtained with and without protective gear. Figure 6 shows the ionizing radiation levels at the 4 different heights in the IR theatre. Figure 7 represents the fluence as a function of the height, 50 cm away from the X-ray tube for an angle of 105° without protection gear. The energy spectrum is given for each of the four heights, i.e. eye lens, chest, belt, and knee.

## Discussion

The available data on direct measurements of the scattered radiation field in IR is sparse. An overview of the literature indicates that almost all the studies are done with Monte Carlo simulations^13–15^. Our measurements provide information on the radiation fluence in the theatre as well as the energy spectrum of the scattered photon field. Those two combined pieces of information are helping to understand the exposure level of the medical staff and to evaluate the homogeneity of the field to which the different people are exposed. This approach is part of the needs of the ICRP and ICRU for fluence-based dosimetry^7^. The spectra presented here clearly show that we should need consideration of energy dependence of dosimeter to evaluate doses (Figs. 3 and 4). Since the X-ray spectrum changes depending on the measurement positions (Figs. 3 and 4), it is important to pay attention to the estimation procedure when attempting to estimate the exposure dose at other locations using the value of the exposure dose measured at a given position. Studying the position of the dosimeter is crucial to avoid underestimation of the dose measurement.

The energy threshold for the Timepix3 is 3 keV due to the calibration curve. Marshall et al.^27^ measured the energy spectrum with a germanium detector with a higher detection threshold set at 20 keV, and Kalyvas et al. obtained a higher threshold set at 10 keV with an Amptek XR-10 CdTe^28^.

The person in position A is the most exposed to ionizing radiations because of his or her proximity to the source of scattered radiation (*i.e.* the entry point of the primary beam in the patient body), even if standing right behind the protective shields (Fig. 3). The least exposed person is the one standing in the back of the theatre, at position D in Fig. 3.

In Fig. 4, the person at position A is less exposed at the level of the eye lens compared to the rest of the body. This can be explained by the fact that the person stands right behind the ceiling protective panels. Collected data also helps to highlight a shift in energy from head to toe at position A, indicating a non-homogenous exposure of the radiologist. The lower exposure level is observed at the level of the eye lens and the shift in energy indicates that if the eye lens dosimetry is performed using a dosimeter worn on the chest, the dose might be overestimated. A dedicated dosimeter would thus be recommended for a better monitoring.

Energy spectra show a more homogeneous exposure for staff standing in the back of the theatre, the spectra shape and fluence are very similar for eye lens, chest and belt. This can be explained by the fact that people stand farther away from the patient who generates the scattered radiation field. For those people, a dosimeter worn on the chest would provide a measurement that is representative of the whole body and of the eye lens exposure. Measured fluence above 74 keV can be due to pile up effect.

The highest exposure for position A and D is observed at the level of the knee. The same pattern was observed by Rehn^29^ with a higher dose close at the knee level when using a C-arm tube, with the tube under the patient couch. An area with a higher scatter exposure rate is found close to the patient, in accordance with Fig. 6. The exposure of staff across a theatre shows that a dosimeter worn at chest level can overestimate the exposure of other organs (e.g. position C) or underestimate it (position A and B). The majority of workers are far from the annual dose limits. On the other hand, this could be a problem for a small fraction of workers, notably radiologists and cardiologists, who may reach dose limits on a dosimeter worn on the chest. Moreover, the articles of Ciraj-Bjelac et al*.*^30^ and Hupe et al*.*^31^ show that performance of many detectors decrease by using in pulsed field.

For pedagogical purposes, we investigated the effectiveness of the radiation protection gear. The use of a lead screen as well as a lead table shield reduces by more than a factor 130 the ionizing radiation field at chest level of a person standing 50 cm away from the tube at an angle of 105°. A spectrum hardening due to absorption by the protective material is clearly observed with a shift from a mean energy of 44 keV without the gear to 50 keV with gear (Fig. 5). Mori and McCaffrey^32,33^ show that this difference in the mean energy can lead to a 12% higher X-ray transmission rate of protective aprons. Figure 5 was of particular interest when we presented the results to the staff and raised questions. It helped to increase awareness and understanding about radiation protection principles. In fact, the medical staff were interested to know how they could adapt their personal radiation protection gear, their positions in the room, or how to move or adapt the collective radiation protection gear. Medical staff were able to see and quantify the effectiveness of the protection, or the most irradiating position in the room. The radiation protection training program for the staff in IR in Lausanne University Hospital uses the DoseAware tool, and it is also very much appreciated by all participants. The Timepix chip shows great potential for offering further options and bringing additional information compared to other pedagogical tools available on the market. The Timepix3 chip provides information on the energy spectrum and opens possibilities to strengthen understanding of effective radiation protection. However, spectra presented are those measured by the sensitive layer of the detector and are not the energy spectra of the scattered radiation field in air. Full deconvolution of the spectrum will be needed before to relate to a dose. Experiences in classrooms have already demonstrated that the Timepix chip could be a valuable educational tool for learning about radiation^34,35^. Our experience demonstrates that the use of the chip as a pedagogical tool can be extended to the medical environment.

All data collected in the theatre was used to generate a coloured distribution map depicting the exposure levels in horizontal planes at four heights. As expected, the highest exposure levels were, for all four planes, next to the X-ray tube. The maximum exposure level was recorded on the horizontal plane at 135 cm (at chest level) on the left side of the tube, i.e. the side that is not covered by the protective gear. The radiologist stands on the right side, behind the gear. The exposure is higher on the two planes under the patient’s bed where the tube is placed, as illustrated on the training video of the Federal office of public health in Switzerland^36^. The radiation distribution represents a specific distribution for the theatre in which we did the measurements and is an innovative and useful tool for the local radiation protection officer. The limitation of this study is the lack of measurements for other angles of the X-ray system.

Figure 7 shows that exposure to the body is height-dependent, therefore varies for the organs. The highest exposure for a person standing on the red star (see Fig. 2, right) is found at chest level. The shapes of the energy spectra are also different with the height. The maximum energy of the scattered radiation is higher at higher heights. Also, if the fluence for the eye lens is lower than for the rest of the body, the mean energy is higher. This information can be important since currently a chest dosimeter is considered sufficient to estimate eye lens exposure. But from this study we noticed the difference between all the energy spectra for different body parts. This confirms that the choice of the dosimeter position is important for an efficient dosimetry surveillance program. A dosimeter worn on the chest may not be representative of the eye lens exposure because of a different radiation exposure level and different energy spectrum.

If we consider the current dosimeters frequently used: TLD and OSL, their response in energy is not constant. TLDs present an energy response ranging from 0.8 to 1.5 up to 1000 keV^37^, while OSL has a linear decreasing response (from 1.07 to 0.85) between 0 and 120 keV^38^. The energy chosen for the calibration of the detector is important, as it is often very different from the energy received monthly. A correction factor must be taken into account^39^. However, this factor is based on an average theoretical energy received and not on the real exposure. In this sense, the Timepix3 brings a new performance and makes it possible to directly calibrate the detector for every possible energy.

Also, the energy correction factor may be different for the chest and the eye lens. A dedicated dosimeter may thus be recommended for eye lens exposure monitoring. Moreover, the radiation field becomes more homogenous the further from the patient while the dose rate decreases. The information collected with the Timepix3 chip in the theatre may also be important with respect to the new approach for dosimetry based on direct conversion coefficients given to the operational quantities from the fluence and energy. This provides us with new parameters for the radiation protection of medical staff.

## Conclusion

Here we have demonstrated the feasibility of using the energy-resolving hybrid pixel detector, Timepix3, to measure X-ray information and characterize the scattered radiation field in an IR theatre. The radiation fields were characterised by measuring the fluence and energy spectra at different positions in an IR theatre. This new approach makes it possible to compare the different energy spectra to which staff members are exposed and thus guide the radiation protection officer in choosing appropriate radiation protection gear. As expected, the radiation fields to which members are exposed vary significantly with their position in the room. Moreover, for a given person, this new radiation detection technique provides information regarding the part of the body that is most exposed. The detector could also be used to correct practitioner practices, for example by accurately positioning the lead screens to improve their protection. Finally, the Timepix3 has demonstrated a great potential as a pedagogical tool to enhance understanding of the ionizing radiation hazards in an interventional theatre.
